# Association between low pH and unfavorable neurological outcome among out-of-hospital cardiac arrest patients treated by extracorporeal CPR: do not dismiss confounders!

**DOI:** 10.1186/s40560-020-00461-4

**Published:** 2020-06-22

**Authors:** Romain Jouffroy, Benoît Vivien

**Affiliations:** SAMU de Paris, Service d’Anesthésie Réanimation, Hôpital Universitaire Necker - Enfants Malades, Assistance Publique - Hôpitaux de Paris, AP-HP, Centre and Université de Paris, Paris, France

**Keywords:** Out of hospital cardiac arrest, pH, ECPR, Outcome, Confounders

## Abstract

Recently, Okada et al. reported an association between low pH value before the implementation of extracorporeal cardiopulmonary resuscitation (ECPR) and 1-month unfavorable neurological outcome among out-of-hospital cardiac arrest (OHCA) patients treated with ECPR.

Nevertheless, we believe that some methodological flaws deserve their conclusions.

The time duration between OHCA occurrence and blood gas analysis (BGA), a major confounder for misinterpretation, was not taken into account. It is not reported whether the result of BGA analysis was considered and/or treated, and if ECPR implementation decision had been influenced by the results analysis. Furthermore, the no-flow duration and the in-hospital phase confounders for neurological outcome are not included as covariates in the logistic regression. Therefore, we believe that causes and consequences should not be confused: the longer is the no-flow duration, the greater are the metabolic consequences.

To the editor:

In the May issue of the Journal, Okada et al. [1] reported in a multi-institutional observational study an association between low pH value (< 7.03) before the implementation of extracorporeal cardiopulmonary resuscitation (ECPR) and 1-month unfavorable neurological outcome among out-of-hospital cardiac arrest (OHCA) patients treated with ECPR. They also suggested that pH value may be helpful to consider the candidate for ECPR.

Undoubtedly, the authors should be congratulated for their noteworthy study on question at the utmost importance. Nevertheless, we do think that their result interpretation requires some words of caution.

Firstly, the authors do not take into account the time duration between OHCA occurrence and blood gas analysis (BGA), which is a major confounder for misinterpretation. Among the 3 tertiles of patients based on the pH value in the BGA, as compared with tertile 1, the median duration is 4.5 min longer for tertile 2 and 8.5 min longer for tertile 3. Such time differences may by themselves fully explain a part of differences in acidaemia depth between the 3 tertiles. Secondly, dividing the pH value into 3 tertiles with equivalent sizes assumes that the relation between pH value and survival is linear, which does not reflect pathophysiology [2]. Thirdly, the authors do not report whether the result of BGA analysis was taken into account in the care delivered decision-making. For example, it is not reported how acidaemia treatment was considered and/or treated, and if ECPR implementation decision had been influenced by the results analysis. Fourthly, from a methodological point of view, the variables included in the multivariate analysis (sex, age, witness of collapse, bystander CPR, prehospital initial rhythm, and initial rhythm on hospital arrival) do not consider the no-flow duration [3] and the in-hospital phase confounders for neurological outcome [4]. Last but not least, ROSC proportions on hospital admission, i.e., before ECPR start, are very different between the tertiles 1, 2, and 3 (respectively 16.3%, 6.8%, and 2%). Thus, we cannot exclude that the higher pH value observed in tertile 1 is not just due to the greater proportion of ROSC in this group that is associated with lower metabolic disturbances.

Therefore, we believe that causes and consequences should not be confused: the longer is the no-flow duration, the greater are the metabolic consequences [5]. Beyond this, the authors’ work is very interesting, but more information and conclusions could be drawn by, first, integrating the no-flow duration in the analysis, and second, using the pH value as a continuous variable in the logistic regression.

## Data Availability

Not applicable

## Abbreviations

BGA: Blood gas analysis
ECPR: Extracorporeal cardiopulmonary resuscitation
OHCA: Out-of-hospital cardiac arrest
ROSC: Return of spontaneous circulation
